# Male Infertility: A Review of Key Papers Appearing in the Reproductive Medicine and Andrology Section of the *Journal of Clinical Medicine*

**DOI:** 10.3390/jcm12062366

**Published:** 2023-03-19

**Authors:** Ettore Caroppo, Giovanni M. Colpi

**Affiliations:** 1 ASL Bari, Reproductive Unit, Andrology Outpatients Clinic, Conversano, 70014 Bari, Italy; 2 Andrology Unit, Procrea Institute, 6900 Lugano, Switzerland

Male infertility accounts for 30% of infertility cases and its prevalence in the general population approximately ranges between 9 and 15%, according to the available surveys [1]. Male infertility is emerging as an important cause of infertility worldwide; the growing scientific interest in this field of reproductive medicine is demonstrated by the increasing number of scientific papers published by journals dedicated to andrology, urology, and reproductive medicine. Since 2020, the *Journal of Clinical Medicine* has dedicated a novel section to Reproductive Medicine and Andrology, with 136 articles being published to date. This editorial aims to highlight the most prominent contributions to the field of male infertility provided by this section of the journal.

Recent research has improved our understanding of the sperm contribution to fertilization and embryo development, which goes well beyond the simple provision of the paternal haploid genome to the oocyte. Chiocchiarelli and coworkers provided an in-dept review of the contribution of histone post-translational modifications (PTMs) to key spermatogenic events, such as the self-renewal and commitment of spermatogonia, meiotic recombination, chromatin remodeling, as well as of their role in sperm function and embryo development. Since many histone PTMs remain associated with the paternal genome during de novo chromatin formation occurring after fertilization, the authors provide some intriguing hypotheses to explain how sperm histones may play a key role for a proper embryonic reprogramming and transgenerational epigenetic inheritance. The authors also reviewed the available evidence on the impact of circular RNAs (circRNAs) on sperm quality and embryo development [2].

Azoospermia due to spermatogenic dysfunction, also termed non-obstructive azoospermia (NOA), is the most severe form of male infertility, affecting about 1% of the male population and 10–15% of the infertile men. A Special Issue in this journal (mdpi.com/journal/jcm/special_issues/NOA_Management) was dedicated to this argument. As more extensively detailed in the accompanying editorial [3], the Special Issue was built on the contributions of opinion leaders such as Peter N. Schlegel and his group, who illustrated how to manage patients with NOA and optimize the success of mTESE [4], as well as the reproductive chances of men with NOA according to the underlying etiologies [5]; Sandro Esteves and coworkers, who provided a detailed explanation on how to discriminate the two forms of azoospermia, obstructive vs. non-obstructive [6]; Krausz and Cioppi, who reviewed the most common genetic abnormalities in men with NOA and their possible impact on their general and reproductive health, as well as on their offspring health [7]; Goulis and colleagues, who showed how the eventual post-surgical hypogonadism may depend upon some clinical factors, including etiology of NOA, the number of previous surgical attempts, and testicular volume [8]; Aydos, who provided an in-depth review of the available procedures to select testicular sperm for ICSI [9]; Smith, who explored the possible application of microfluidic technology to isolation of sperm from testicular samples [10]; and our group, who proposed a detailed description of the microTESE surgical technique [11], reviewed the current evidence about the possible effect of hormonal treatments on the outcome of surgical sperm retrieval [12], and evaluated the studies attempting to individuate reliable predictors of sperm retrieval outcome [13].

The severe spermatogenic dysfunction in patients with NOA may be due to several potential causes; however, in most cases, a clear etiology cannot be identified. A pilot study published in the present section of the journal proposed that patients with NOA may have an altered microbiota compared to healthy males [14]. Using shotgun metagenomic sequencing and analysis on plasma and fecal samples obtained from healthy males and patients with NOA, the authors found gut microbiota dysbiosis in patients with NOA. Beneficial gut microbes such as Ruminococcus bicirculans and B. thetaiotaomicron were less abundant, while bacteroides Bacteroides vulgatus and Streptococcus thermophilus were more abundant in these patients compared to healthy men. The authors hypothesized that such dysbiosis may be associated with chronic inflammation, endothelial damage, and blood–testis barrier disruption, which could be responsible for spermatogenic damage. Such a provocative hypothesis needs to be confirmed by further studies.

Despite the severe spermatogenic impairment that characterizes patients with NOA, sperm may be retrieved in more than half of cases; in the remaining cases, however, no other option than donor sperm IVF is currently available. The in vitro maturation of early-stage germ cells could be a promising treatment option for these patients; however, obtaining functional spermatozoa from human spermatogonia is not actually feasible. Nevertheless, researchers are developing many different culture systems for the in vitro maturation of early-stage germ cells. Kaan Aydos and colleagues explored the feasibility of co-culturing the testicular tissue of patients with NOA with healthy Sertoli cells derived from men with obstructive azoospermia, whose spermatogenesis is not compromised, in a medium supplemented with FSH and testosterone. They first assessed that healthy Sertoli cells could be easily cryopreserved, and that the freezing–thawing process did not activate apoptotic mechanisms in these cells. Afterwards, tissue samples obtained from patients with NOA were cultured in two distinct environments containing FSH and testosterone, or FSH, testosterone plus co-culture with healthy Sertoli cells. The results show that co-culture with FSH and testosterone was an effective treatment for germ cell maturation, while adding Sertoli cells from healthy individuals was insignificant compared to using the intact Sertoli cells retrieved from the patients’ own testes [15].

Fertility preservation by means of immature testicular tissue may represent a fertility preservation strategy in prepubertal boys with cancer. An interesting experimental study performed thanks to collaboration between the Centre for Reproductive Health of the University of Edinburgh, the Research Laboratory for Reproduction, Genetics and Regenerative Medicine, Vrije Universiteit Brussel, and the NORDFERTIL Research Lab of the Karolinska Institute and Karolinska University Hospital demonstrated that exogenous human chorionic gonadotropin stimulation was able to maintain the Leydig cell steroidogenesis, the acquisition of features of Sertoli cell maturation, and blood–testis barrier development, none of which were present prior to the transplantation, by human fetal testis transplanted subcutaneously into castrated immunocompromised mice [16].

Finally, we would like to draw the attention of the readers of the *JCM* to an interesting review on the hormonal treatment of male infertility. The follicle-stimulating hormone (FSH) is effective in promoting spermatogenesis and inducing fertility in patients with FSH deficiency; however, such a therapeutical strategy has been adopted in the past three decades for infertile men with normal pituitary function to improve spermatogenesis and, consequently, to increase their chances of becoming biological fathers. The group of the University of Modena, Italy, led by Prof. Manuela Simoni, a renowned expert in the field, reviewed the physiological role played by FSH in spermatogenesis and its potential therapeutic action in men. The authors exposed the mechanism of FSH action in the Sertoli cell and the genetic regulation of FSH action on spermatogenesis, and provided evidence for the possibility of spermatogenesis hyperstimulation in normogonadotropic infertile men. Still, they remarked that the most effective dose and duration of FSH treatment have yet to be determined [17].
